# In Vitro Antiviral Properties of Two Recombinant Sendai Virus Vectors Encoding *ORFV 011* and *ORFV 059* Genes

**DOI:** 10.3390/v18040462

**Published:** 2026-04-13

**Authors:** Álex Gómez, Idoia Glaria, Irati Moncayola, Leonor Puzol, Laura Arriazu, Ainhoa Calero, Ignacio de Blas, Mikel Nazábal, Itziar Hualde, Benhur Lee, Lluís Luján, Ralf Amann, Irache Echeverría, Ramsés Reina

**Affiliations:** 1 Departamento de Patología Animal, Universidad de Zaragoza, 177 Calle de Miguel Servet, 50013 Zaragoza, Spain; a.gomez@unizar.es (Á.G.); deblas@unizar.es (I.d.B.); lluis.lujan@unizar.es (L.L.); 2 Instituto Agroalimentario de Aragón-IA2, Universidad de Zaragoza, 177 Calle de Miguel Servet, 50013 Zaragoza, Spain; 3 Instituto de Agrobiotecnología (CSIC-Gobierno de Navarra), 123 Avenida Pamplona, 31192 Mutilva, Spain; idoia.g@csic.es (I.G.); irati.moncayola@csic.es (I.M.); leonor.puzol@csic.es (L.P.); laura.arriazu@csic.es (L.A.); ainhoacalero1@gmail.com (A.C.); 4 Instituto Navarro de Tecnologías e Infraestructuras Agroalimentarias (INTIA), 22 Avenida Serapio Huici, 31610 Villava/Atarrabia, Spain; mnazabal@initiasa.es (M.N.); ihualde@intiasa.es (I.H.); 5 Department of Microbiology, Icahn School of Medicine at Mount Sinai, 1 Gustave L. Levy Pl, New York, NY 10029, USA; benhur.lee@mssm.edu; 6 Department of Immunology, Interfaculti Institute for Cell Biology, University Hospital Tübingen, 15 Auf d. Morgenstelle, 72076 Tübingen, Germany; ralf.amann@ifiz.uni-tuebingen.de; 7 Departamento de Agronomía, Biotecnología y Alimentación, Universidad Pública de Navarra, s/n Avenida Barañain, 31008 Pamplona, Spain; irache.echeverria@unavarra.es

**Keywords:** Orf virus, Sendai virus, viral vector, innate immunity, antiviral

## Abstract

Orf virus (ORFV) is a globally distributed zoonotic parapoxvirus that causes a highly contagious mucocutaneous disease in small ruminants. Despite the urgent demand for vaccination-based control, no licensed vaccines are currently available universally. In this study, we generated two recombinant Sendai virus (SeV) vectors expressing *ORFV 011* (rSeV-GFP-B2L) and *ORFV 059* (rSeV-GFP-059) genes and evaluated their ability to stimulate antiviral responses in vitro. Following the transduction, we assessed transgene expression, innate immune activation, induction of interferon-stimulated genes (*A3Z1*, *OBST2*, *SAMHD1*), and antiviral activity. Both vectors significantly upregulated pattern recognition receptors (*TLRs*, *RIG-I*) and type I interferon (*IFN-β*) genes, with rSeV-GFP-059 inducing the strongest response. Remarkably, *OBST2* was robustly upregulated, suggesting a potential role in restricting ORFV replication. Antiviral activity assays revealed a marked reduction in ORFV DNA copies and a mild decrease in ORFV RNA transcription in rSeV-GFP-059-transduced cells, particularly at later time points, accompanied by complete abrogation of the typical cytopathic effect. Collectively, these results demonstrate that SeV-based vectors, particularly rSeV-GFP-059, efficiently prime antiviral immunity and suppress ORFV replication, establishing a promising platform for further in vivo vaccine evaluation in sheep.

## 1. Introduction

Contagious ecthyma (CE), caused by the Orf virus (ORFV), is a highly contagious zoonotic viral mucocutaneous disease that primarily affects sheep and goats worldwide [1,2,3]. CE hinders lamb feeding, decreasing their daily weight gain and increasing the sanitary and personnel costs per animal, resulting in significant global economic losses [4,5,6]. CE is characterized by multifocal vesiculopustular and/or proliferative dermatitis affecting the muzzle, nipples and limbs, as well as multifocal erosive–ulcerative stomatitis, esophagitis and/or ruminitis [3,7]. CE lesions are usually contaminated by bacterial or fungal agents, which further complicates clinical progression [8].

ORFV, a member of the family *Poxviridae*, subfamily *Chordopoxvirinae*, and genus *Parapoxvirus* [9], is an enveloped virus characterized by a double membrane that surrounds the double-stranded DNA (dsDNA) genome containing 132 genes in approximately 135–140 kilobases [10,11]. Conserved genes, located in the center of the genome, are responsible for replication and transcription [12]. Virulence and pathogenicity genes (accessory genes) are found at both extremes, adjacent to the inverted terminal repeats [12,13,14]. Accessory genes encode several immunomodulatory proteins that enable the virus to evade the immune response [15,16,17], thereby complicating disease control [18].

Although the development of novel, safe, and effective vaccines for ORFV is a priority, no universally licensed vaccines for sheep and goats are currently available [3,18,19]. Purified scab-based vaccines were used in the 1930s because they induced an effective and long-lasting protection [19,20]. However, these non-attenuated live virus vaccines do not confer cross-protection [21] and induce vaccine-associated ORFV cutaneous lesions [22], especially among unvaccinated animals [19]. Culture-based live-attenuated vaccines are relatively safer [19,23,24]; therefore, in some countries, vaccines such as Scabivax^®^ Forte (MSD Animal Health, Milton Keynes, UK) or ECTHYBEL^®^ (Boehringer Ingelheim Animal Health France, Lyon, France) have been approved and commercialized. However, these types of vaccines can revert to virulence [25] or be contaminated [26], elicit only partial [25,27] and short-lived (3–6 months) immunity [19,23,28,29], and the transmission of protection to lambs has not been demonstrated [30]. They are only recommended in enzootic flocks to reduce clinical severity [23]. A double gene-deleted recombinant vaccine (rGS14-DCBP-DGIF) with deletions in Chemokine-Binding Protein (*CBP*) and *GIF* genes; a triple gene-deleted mutant of ORFV (rGS14∆CBP∆GIF∆121) with deletions in *CBP*, *GIF* and *ORFV 121* genes; and a quadruple gene-deleted mutant with an additional deletion in viral IL-10 gene (*vIL-10*) (rGS14∆CBP∆GIF∆121∆VL10) showed complete protection against virulent ORFV challenge in kids [31,32,33]. Other safer strategies include prototypes of subunit and plasmid DNA vaccines that are based on the highly conserved central immunogenic envelope proteins ORFV B2L (*ORFV 011* gene) and ORFV F1L (*ORFV 059* gene) [34,35]. ORFV B2L is considered the homolog of the poxviral phospholipase protein (F13) and Vaccinia virus envelope protein antigen-p37K, responsible for extracellular virus formation [14,36,37]. ORFV F1L is homologous to the Vaccinia virus *H3L* gene [38], and is therefore probably involved in morphogenesis and viral infection [39], and it is responsible for the induction of neutralizing antibodies [39,40,41]. These types of vaccines have been explored prophylactically in mice, achieving complete protection against ORFV challenge [37,42,43,44,45]. Nevertheless, experimental trials have not been carried out in small ruminants.

Currently, viral vector-based vaccines are extensively studied for their immunogenic potential [46,47,48] and their efficiency in transgene expression [49,50]. A Vaccinia-based vector expressing multiple fragments of ORFV DNA demonstrated significant protection against wild-type ORFV challenge in lambs; however, the specific immunogenic ORFV proteins were not identified [51]. Sendai virus (SeV) has shown promising safety and efficacy as a viral vector in developing vaccine prototypes for various viral pathogens [48,52,53]. In sheep, SeV has been effective in transgene expression [49,50] and robustly activated the innate immune response in sheep cells by upregulating the expression of interferon-stimulated genes (ISGs) expression, including apolipoprotein B mRNA editing enzyme catalytic subunit 3 (*APOBEC3*/*A3Z1*), ovine BST2/Tetherin (*OBST2*) and SAM and HD domain containing deoxynucleoside triphosphate triphosphohydrolase 1 (*SAMHD1*), all of which are involved in in vitro and in vivo protection against small ruminant lentiviruses (SRLV) [54,55]. Therefore, recombinant SeV vectors could represent an alternative strategy for developing a new generation of safe and effective vaccines against ORFV infection in small ruminants.

In this study, we developed two recombinant SeV vectors encoding *ORFV 011* (rSeV-GFP-B2L) and *ORFV 059* (rSeV-GFP-059) genes and evaluated the transgene expression, activation of the innate immune response and antiviral activity against ORFV and SRLV in vitro.

## 2. Materials and Methods

### 2.1. Cells, Plasmids, and Viruses

Ovine skin fibroblasts (OSF) were obtained from skin biopsies of ORFV-free lambs. These animals tested negative, using an in-house ORFV-specific indirect enzyme-linked immunosorbent assay (ELISA) based on the ORFV 109 protein and polymerase chain reaction (PCR) targeting the *045 ORFV* gene [7,56] (Supplementary Table S1). OSF, human embryonic kidney (HEK293T) cells and bovine esophagus (KOP-R) cells were cultured at 37 °C with 5% CO_2_ in Dulbecco’s modified Eagle’s medium (DMEM) (Deltalab, Rubí, Spain) supplemented with 10% heat-inactivated fetal bovine serum (FBS), 1% L-glutamine and 1% antibiotic/antimycotic mix (Sigma Aldrich, St. Louis, MO, USA).

The SeV antigenome plasmid containing green fluorescent protein (GFP) (SeV-GFP), along with accessory plasmids (T7-SeV-N, T7-SeV-P, T7-SeV-L and T7opt), were used as previously described [57]. SeV-GFP derives from the SeV Fushimi strain, with mutations introduced into the *F* and *M* genes, as previously described [58].

The ORFV strain NAV (GenBank Accession no. ON805832) was isolated from proliferative scabby skin lesions of *Assaf* breed lambs during a natural CE outbreak in Navarra (Spain), as described previously [7,59]. For virus propagation, ORFV strain NAV was cultured in KOP-R cells, and supernatants were collected and filtered once approximately 90% of the culture showed a cytopathic effect (CPE). Viral titers were determined on 96-well KOP-R cell cultured plates using the Reed–Müench method [60] and expressed as a 50% tissue culture infectious dose per milliliter (TCID_50_/mL). SRLV viral stocks from genotype A (strain EV1) [61] were propagated and titrated in OSF, as described above.

### 2.2. Construction and Viral Rescue of Recombinant SeV Plasmids

*ORFV 011* (1206 base pairs (bp)) and *ORFV 059* (1029 bp) gene sequences were amplified from the ORFV strain NAV and cloned into the SeV-GFP plasmid by In-Fusion^®^ cloning technology, between *Gaussia-Dura Luc* and *GFP* genes (primers in Supplementary Table S1; In-Fusion HD Cloning Kit; Takara Bio USA, San Jose, CA, USA), generating recombinant plasmids rSeV-GFP-B2L and rSeV-GFP-059. Correct insertion was checked by Sanger sequencing.

Recombinant viruses were rescued using the SeV reverse genetics system [57]. Briefly, 0.5 µg of antigenomic SeV-GFP, rSeV-GFP-B2L or rSeV-GFP-059, along with the accessory plasmids (0.173 µg T7-SeV-N, 0.1 µg T7-SeV-P, 0.01 µg T7-SeV-L, 0.5 µg of T7opt) were co-transfected in 60–70% confluent HEK293T cells using Jet Prime transfection reagent (jetPRIME^®^; Polyplus, Illkirch, France), following the manufacturer’s instructions (1:2, DNA:transfection reagent ratio). Transfection efficiency was monitored by fluorescence microscopy (Nikon Eclipse TE300; Nikon Corporation, Tokyo, Japan) to detect virus-encoded GFP expression. GFP-positive supernatants were collected at 96 h post-transfection, clarified by centrifugation at 1400 g for 5 min, filtered and stored at −80 °C.

Viral stocks were amplified by five successive passages in HEK293T cells at a multiplicity of infection (MOI) of 1 and titrated by fluorescence microscopy in 96-well culture plates, using the Reed–Muench method [60].

### 2.3. Quantification of mRNA Relative Expression in OSF

OSF were transduced with SeV-GFP, rSeV-GFP-B2L or rSeV-GFP-059 at a MOI of 1. Cell lysates were collected at 12, 24, 48, 72, 96 and 120 h post-transduction (hpt). mRNA extraction was performed using an automated system (NucleoMag RNA-Magnetapure 32; Dominique Dutscher, Bernolsheim, France), and cDNA was synthesized using random hexamers and oligo (dT) primers (PrimeScript RT Reagent Kit; Takara Bio, Kyoto, Japan). A quantitative PCR (qPCR) was performed using SYBR Premix Ex Taq (Takara, Kyoto, Japan) with specific primers (Supplementary Table S1) on an AriaMx Real Time PCR System (Agilent Technologies, Santa Clara, CA, USA).

Expression of *ORFV B2L* and *ORFV 059* transgenes was evaluated at 24, 48, 72, 96 and 120 hpt. β-actin was used as the housekeeping gene for mRNA quantification (2^−ΔCt^ method). To assess innate immune stimulation by SeV-GFP, rSeV-GFP-B2L or rSEV-GFP-059, the upregulation of different Toll-like receptors (*TLRs*), retinoic acid-inducible gene I (*RIG-I*), adaptor MyD88 and interferon β (*IFN-β*) were measured at 12, 24, 48 and 72 hpt in OSF. MyD88 was included as a target gene to assess innate immune signaling downstream of TLR activation. β-actin was used as the sole housekeeping gene for normalization. Basal gene expression in non-transduced OSF was used as the threshold value for relative quantification (2^−ΔΔCt^ method). Using a logarithmic scale, genes with values higher than 1 were considered to be upregulated.

Additionally, expression of ISGs, including *A3Z1*, *OBST2* and *SAMHD1*, was evaluated at 24, 48 and 72 hpt in OSF, using specific primers (Supplementary Table S1) and the 2^−ΔΔCt^ method.

### 2.4. Antiviral Activity Assays

KOP-R cells were transduced at 0.1 MOI with SeV-GFP, rSeV-GFP-B2L or rSeV-GFP-059. Supernatants were removed at 48 hpt and cells were infected with the ORFV strain NAV at 0.1 MOI. DNA was extracted manually (E.Z.N.A.^®^ Blood DNA Kit; Omega Biotek, Norcross, GA, USA) at 24 h post-infection (hpi) and 5 days post-infection (dpi). The quantity and purity of the extracted nucleic acids were evaluated with a microvolume ultraviolet-visible spectrophotometer (NanoDrop OneC; Thermo Scientific^®^, Waltham, MA, USA). Viral DNA was quantified by qPCR on an AriaMx qPCR system (Agilent Technologies, Santa Clara, CA, USA), applying 50 ng of DNA, SYBR Premix Ex Taq (Takara, Kyoto, Japan) and specific primers targeting the *ORFV 045* gene [7,56] (Supplementary Table S1). A standard curve (R^2^: 0.98) was used to determine the number of ORFV copies per nanogram of DNA (Log_10_ copies ORFV/ng), as described previously [50]. Moreover, mRNA was also extracted and purified as described above at 24 and 48 hpi. The quantification of *ORFV 045* expression was evaluated using the 2^−ΔCt^ method. Additionally, the ORFV-specific CPE was also evaluated at 5 dpi. The positive control consisted of KOP-R cells infected with the ORFV strain NAV.

Supernatants from OSF transduced at 1 MOI with SeV-GFP, rSeV-GFP-B2L or rSeV-GFP-059 were collected and filtered at 24, 48 and 72 hpt. The antiviral activity of these supernatants was evaluated by incubation with fresh OSF cells. After 24 h of incubation, OSF were infected with the SRLV strain EV1 at 0.5 MOI. At 24 hpi, cells were collected and DNA extracted as described above. Viral load was measured by qPCR, using specific primers (Supplementary Table S1). A standard curve (R^2^: 0.98) was used to determine SRLV copies per nanogram of DNA (Log_10_ copies SRLV/ng) with a range of detection from 10^1^ to 10^8^ copies/ng. The positive control consisted of OSF infected with SRLV strain EV1.

### 2.5. Statistical Analysis

Data were analyzed using IBM SPSS 26.0 (IBM Corporation, Armonk, NY, USA). Each in vitro experiment was repeated a minimum of three times. Continuous variables (quantification of mRNA relative expression, copies per nanogram from antiviral activity assays) were described using the mean and standard deviation. After the Shapiro–Wilk test was used to assess the normal distribution of the data, Levene’s test was applied to assess the homogeneity of variance of normally distributed variables. For variables with a normal distribution and equal variances, a one-way analysis of variance (ANOVA) or Student’s *t*-test was applied. After ANOVA analysis, Bonferroni correction was used for multiple pairwise comparisons. For normally distributed variables and unequal variances, Welch’s *t*-test was applied and multiple pairwise comparisons were performed using a Games Howell post hoc test. For non-normally distributed variables, the Kruskal–Wallis test or Mann–Whitney U test was conducted. After the Kruskal–Wallis test, a Dunn post hoc was applied for multiple pairwise comparisons. The statistical significance was set at *p* < 0.050 and represented as * *p* < 0.050, ** *p* < 0.010, and *** *p* < 0.0010.

## 3. Results

### 3.1. Generation and Titration of Recombinant SeV Vectors

**Figure 1:**
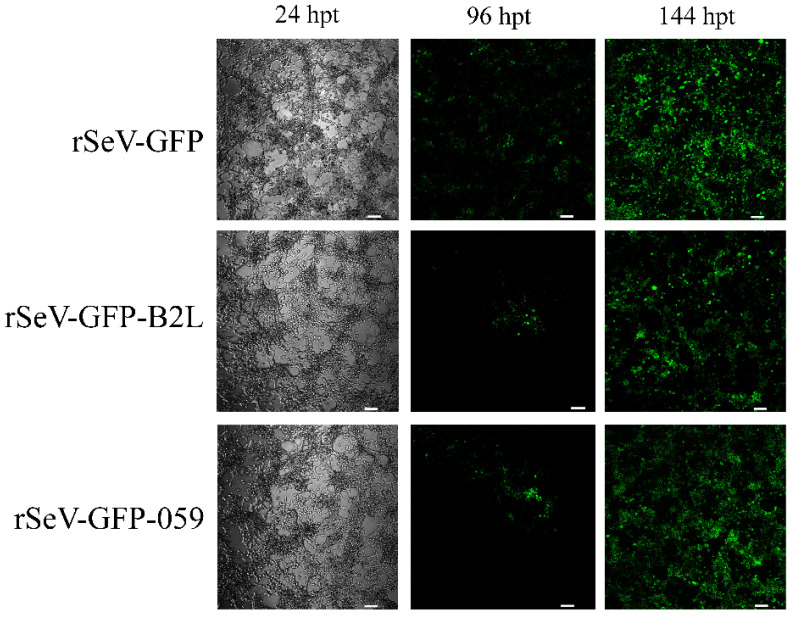
HEK293T cells transfected with SeV-GFP, rSeV-GFP-B2L or rSeV-GFP-059. The transfection efficiency was determined by the presence of GFP-positive cells under fluorescence microscopy at 24, 96 and 144 h post-transfection (hpt). (Scale bar = 15 µm).

The transfection of HEK293T cells with SeV-GFP, rSeV-GFP-B2L or rSeV-GFP-059 was confirmed by the presence of GFP-positive cells under fluorescence microscopy. SeV-GFP transfection resulted in approximately 40–50% of GFP-positive cells at 96 hpt, whereas rSeV-GFP-B2L and rSeV-GFP-059 showed lower transfection efficiency (5–10%). All three recombinant SeV vectors reached approximately 80–100% GFP-positive cells at 144 hpt (Figure 1). Subsequently, SeV-GFP, rSeV-GFP-B2L and rSeV-GFP-059 were collected, filtered and titrated on HEK293T cells, achieving titers of 10^7^ TCID_50_/mL.

### 3.2. Transgene Expression in OSF

**Figure 2:**
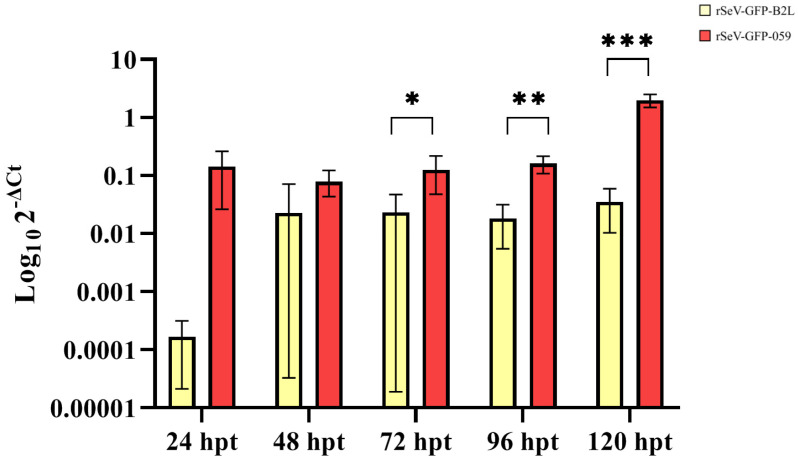
Relative mRNA transgene expression in OSF transduced with rSeV-GFP-B2L or rSeV-GFP-059 at 24, 48, 72, 96 and 120 h post-transduction (hpt). Data represent the mean and standard deviation. Statistically significant differences between groups (* *p* < 0.050, ** *p* < 0.010, *** *p* < 0.001).

Transduction of OSF with the recombinant SeV vectors was confirmed by GFP-positive cells. OSF transduced with rSeV-GFP-B2L and rSeV-GFP-059 showed efficient and progressive transgene expression (Figure 2). OSF transduced with rSeV-GFP-059 showed higher transgene expression than OSF transduced with rSeV-GFP-B2L at 72 hpt (*p* = 0.016), 96 hpt (*p* = 0.001) and 120 hpt (*p* < 0.001). Both viral vectors reached a peak of transgene expression at 120 hpt.

### 3.3. Stimulation of the Innate Immune Response in OSF

**Figure 3:**
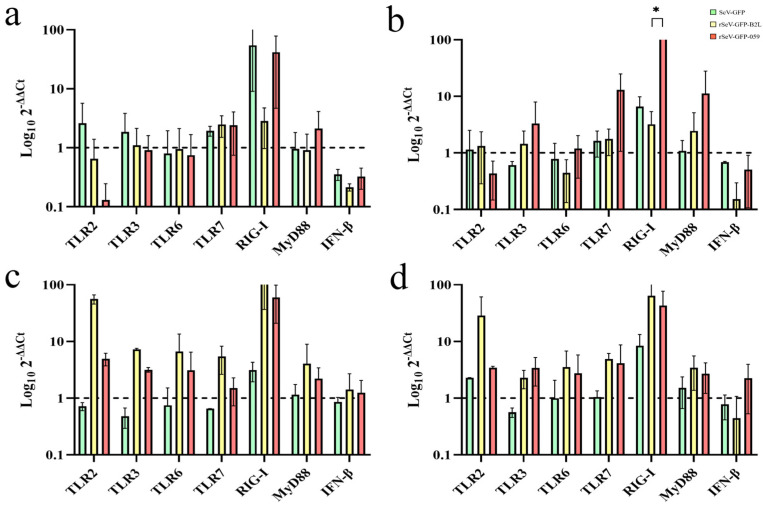
Relative mRNA expression of upregulated *TLRs*, *RIG-I*, *MyD88* and *IFN-β* genes in OSF transduced with SeV-GFP, rSeV-GFP-B2L or rSeV-GFP-059 at 12 (**a**), 24 (**b**), 48 (**c**) and 72 (**d**) hours post-transduction (hpt). Genes with expression values >1 (broken line) were considered to be upregulated. Data represent the mean and standard deviation. Statistically significant differences between groups (* *p* < 0.05).

The mRNA expression of different *TLRs*, *RIG-I*, *MyD88* and *IFN-β* genes varied depending on the SeV vector used and time post-transduction (Figure 3). OSF transduced with rSeV-GFP-B2L and rSeV-GFP-059 showed upregulation of the *TLR2*, *TLR3*, *TLR6*, *TLR7*, *Myd88*, *RIG-I* and *IFN-β* genes. In contrast, SeV-GFP did not induce upregulation of the *TLR6* or *IFN-β* genes. At 24 hpt, rSeV-GFP-059 induced a statistically significant higher upregulation of *RIG-I* than rSeV-GFP-B2L (*p* = 0.039). *RIG-I* was the most upregulated gene, peaking at 12 hpt in OSF transduced with SeV-GFP, at 48 hpt with rSeV-GFP-B2L and at 24 hpt with rSeV-GFP-059.

### 3.4. Interferon-Stimulated Genes Expression in OSF

**Figure 4:**
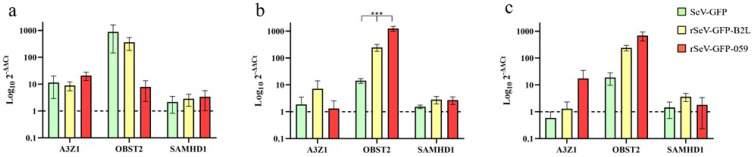
Relative mRNA expression of interferon-stimulated genes (*A3Z1*, *OBST2* and *SAMHD1*) in OSF transduced with SeV-GFP, rSeV-GFP-B2L or rSeV-GFP-059 at 24 (**a**), 48 (**b**) and 72 (**c**) hours post-transduction (hpt). Dotted line corresponds to non-transduced OSF. Data shown are the mean and standard deviation. Statistically significant differences between groups (*** *p* < 0.001).

OSF transduced with SeV-GFP, rSeV-GFP-B2L and rSeV-GFP-059 generally overexpressed ovine ISGs (*A3Z1*, *BST2* and *SAMHD1*) (Figure 4). Transduction with rSeV-GFP-059 induced higher *OBST2* expression than SeV-GFP and rSeV-GFP-B2L at 48 hpt (*p* < 0.001). *OBST2* was the most upregulated ISG (*p* < 0.05), except at 24 hpt in rSeV-GFP-059-transduced OSF, where *A3Z1* was more highly expressed compared to *OBST2* and *SAMHD1* (*p* = 0.011; *p* = 0.024). Additionally, at 48 hpt and 72 hpt *OBST2* expression was significantly higher than *A3Z1* (*p* = 0.032; *p* = 0.026) and *SAMHD1* (*p* = 0.048; *p* = 0.041) in rSeV-GFP-059-transduced OSF and at 72 hpt in rSeV-GFP-B2L-transduced OSF (*p* = 0.003; *p* = 0.018).

### 3.5. Antiviral Responses

**Figure 5:**
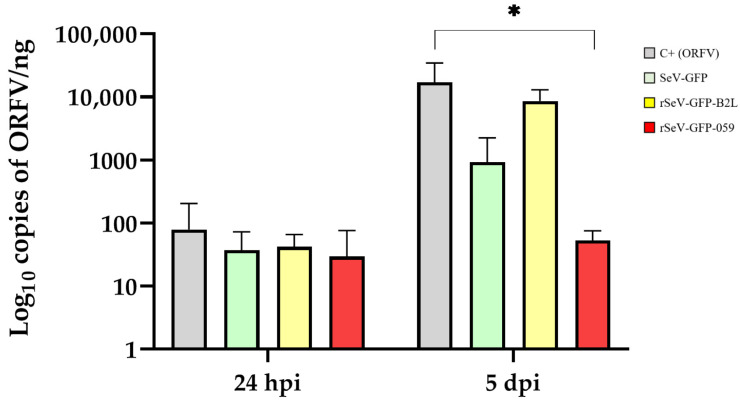
ORFV DNA quantification in KOP-R transduced with SeV-GFP, rSeV-GFP-B2L or rSeV-GFP-059 and infected (48 h post-transduction) with ORFV strain NAV. DNA were quantified at 24 h post-infection (hpi) and 5 days post-infection (dpi). Non-transduced KOP-R cells infected with ORFV strain NAV at the mentioned times served as positive controls (C+ (ORFV)). Data represent the mean and standard deviation. Statistically significant differences between groups (* *p* < 0.05).

**Figure 6:**
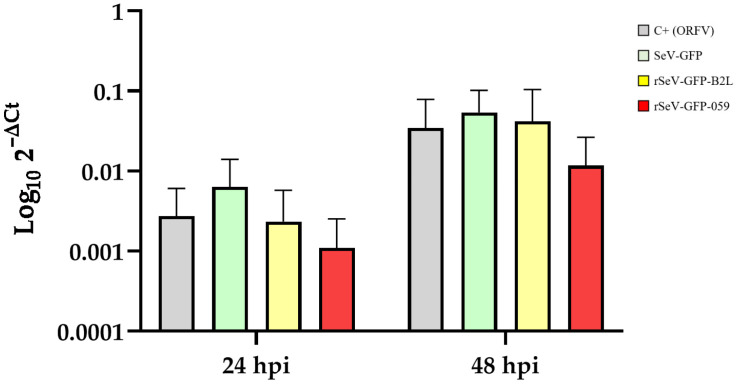
Relative mRNA expression of *ORFV 045* in KOP-R transduced with SeV-GFP, rSeV-GFP-B2L or rSeV-GFP-059 and infected (48 h post-transduction) with ORFV strain NAV. Expression of ORFV 045 was quantified 24 and 48 h post-infection (hpi). Non-transduced KOP-R cells infected with ORFV strain NAV at the mentioned times served as positive controls (C+ (ORFV)). Data represent the mean and standard deviation.

**Figure 7:**
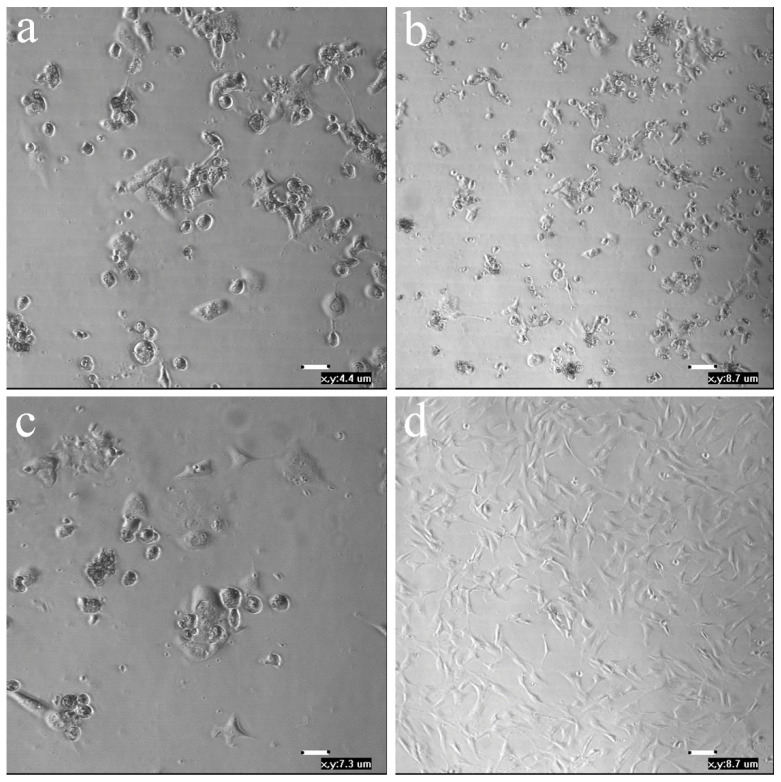
Evaluation of the ORFV-specific cytopathic effect 5 days post-infection with ORFV strain NAV in non-transduced-KOP-R cells (**a**) and KOP-R cells transduced with SeV-GFP (**b**), rSeV-GFP-B2L (**c**) and rSeV-GFP-059 (**d**). The x, y values denote the calibrated pixel resolution in micrometers (µm) for the respective micrographs.

Viral load varied among transduced and non-transduced cells once infected with ORFV. Whereas only a few differences were observed at 24 hpi, a significant decrease in ORFV DNA copies was observed at 5 dpi in KOP-R cells transduced with rSeV-GFP-059 compared to the positive control (*p* = 0.048) (Figure 5). Similarly, although no significant differences were observed, ORFV RNA transcription was reduced at 24 and 48 hpi in rSeV-GFP-059-transduced KOP-R cells (Figure 6). Moreover, typical ORFV-induced CPE was completely abolished in rSeV-GFP-059-transduced KOP-R cells compared to the other groups (Figure 7).

**Figure 8:**
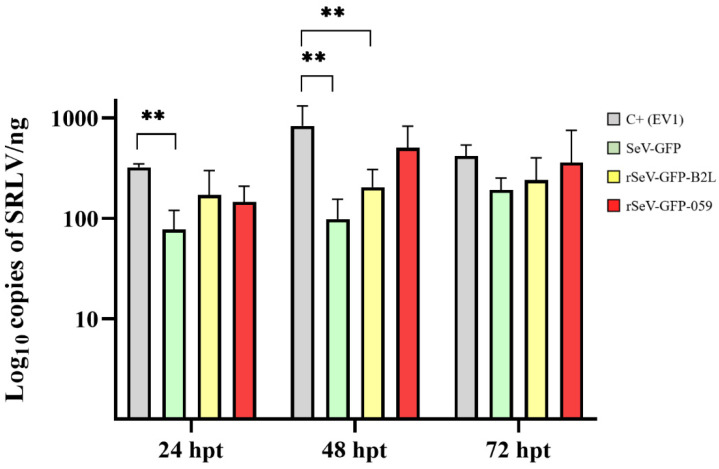
SRLV DNA quantification in OSF incubated with supernatants from OSF transduced with SeV-GFP, rSeV-GFP-B2L or rSeV-GFP-059 and infected with SRLV strain EV1 after 24 h. Supernatants were collected at 24, 48 and 72 h post-transduction (hpt). Non-transduced OSF infected with EV1 at the mentioned times served as positive controls (C+ EV1). Data shown are the mean and standard deviation. Statistically significant differences between groups (** *p* < 0.01).

Supernatants from OSF transduced with SeV-GFP, rSeV-GFP-B2L or rSeV-GFP-059 and collected at 24, 48 and 72 hpt reduced the copy number of SRLV DNA (Figure 8). Specifically, supernatants collected at 24 hpt from SeV-GFP-transduced OSF significantly reduced SRLV DNA copies compared to the positive control (*p* = 0.009). Similarly, supernatants collected at 48 hpt from SeV-GFP or rSeV-GFP-B2L-transduced OSF showed a lower SRLV load than the positive control (*p* = 0.002; *p* = 0.004). Although reductions were observed at 72 hpt, no statistically significant differences were observed.

## 4. Discussion

This study demonstrated the successful generation and rescue of recombinant SeV vectors, using the SeV reverse genetics system [57] followed by titration in HEK293T cells, yielding high viral titers (10^7^ TCID_50_/mL). Recombinant SeV vectors efficiently expressed immunogenic *ORFV 011* (rSeV-GFP-B2L) and *ORFV 059* (rSeV-GFP-059) genes in OSF. Moreover, rSeV-GFP-059 elicited a strong activation of the innate immune response and the upregulation of ISGs and conferred in vitro protection against ORFV, supporting its potential as a promising vaccine candidate against ORFV.

Remarkably, rSeV-GFP-059 induced significantly higher transgene expression than rSeV-GFP-B2L, suggesting that *ORFV 059* may possess intrinsic features that favor transcription, translation or mRNA stability in OSF. Similar results have been reported in ovine turbinate cells after transfection with naked DNA plasmids, where *ORFV B2L* expression was enhanced in the chimeric B2L-059 construct [42].

The innate immune response triggered by these recombinant vectors was robust and gene-specific. Both rSeV-GFP-B2L and rSeV-GFP-059 induced the upregulation of key pattern recognition receptor (PRRs) genes, including *TLR2*, *TLR3*, *TLR6*, *TLR7*, and *RIG-I*, as well as downstream signaling molecules such as *MyD88* and *IFN-β*. In this study, rSeV-GFP-B2L and rSeV-GFP-059 induced higher upregulation of all PRRs at 48 hpt compared to SeV-GFP, suggesting that ORFV B2L and 059 proteins enhance innate immune responses in ovine cells. Interestingly, SeV-GFP alone failed to upregulate *TLR6* and *IFN-β* genes, highlighting the immunostimulatory potential of the ORFV transgenes. TLR2/TLR6 heterodimers are known to detect viral envelope proteins such as paramyxovirus hemagglutinin-neuraminidase [62,63,64], but also other ORFV-related viruses such as *Vaccinia* [65]. This suggests that ORFV transgenes may encode components or induce signals that engage the TLR2/TLR6 pathways, supporting their potential role in ORFV recognition. Interestingly, previous studies suggest that ORFV may be recognized by TLR4, TLR5, TLR7, TLR9, and TLR10 [66,67,68]. However, only upregulation of TLR7 was induced by our recombinant SeV vectors. In this study, *RIG-I* emerged as the most consistently upregulated gene, with distinct temporal expression peaks depending on the vector used, suggesting differential kinetics of innate immune activation. TLR3 and RIG-I signaling pathways have been shown to induce antiviral defenses in keratinocytes [69], suggesting that these recombinant viruses may provide initial protection against ORFV infection. A direct comparison between the recombinant viruses showed no statistically significant differences, except in OSF transduced with rSeV-GFP-059, where *RIG-I* expression was significantly higher than in rSeV-GFP-B2L-transduced OSF at 24 hpt. RIG-I is a cytosolic PRR that plays a key role in detecting short double-stranded RNA (dsRNA) or single-stranded RNA (ssRNA) with a 5′-triphosphate (5′ppp) group, features that are commonly found in non-self viral RNA. Sendai virus, being a negative-sense ssRNA virus, produces replication intermediates and uncapped 5′ppp RNA during its life cycle, which are potent activators of RIG-I. The upregulation of RIG-I in OSF transduced with SeV vectors, especially rSeV-GFP-059, suggests that SeV RNA itself, or the expression of ORFV transgenes, may enhance the production of RIG-I ligands, thereby amplifying innate immune signaling. Although ORFV is a dsDNA virus and not a direct ligand for RIG-I, the expression of ORFV genes within RNA viral vectors may enhance their innate immune activation. These results suggest that recombinant SeV vectors not only serve as delivery platforms, but also act as immunostimulatory agents that are capable of engaging cytosolic sensors such as RIG-I, thereby enhancing their vaccine potential.

The *IFN-β* gene was upregulated exclusively in OSF transduced with rSeV-GFP-B2L at 48 hpt and with rSeV-GFP-059 at both 48 and 72 hpt. Since rSeV-GFP-B2L and rSeV-GFP-059 induced higher upregulation of *TLRs* and *RIG-I* than SeV-GFP, higher *IFN-β* could be expected, as observed. Although SeV is generally recognized as a potent inducer of type-I IFN [70,71,72], the SeV-GFP variant used in this study is derived from the Fushimi strain and carries mutations in the *F* and *M* genes [57], which reduce its infectivity and replication capacity. These modifications likely result in a weaker stimulation of *IFN-β* expression. Importantly, type I IFN responses are known to inhibit viral replication through the upregulation of the ISGs [50,54].

The expression of ISGs further confirmed the immunostimulatory capacity of the recombinant SeV vectors. *OBST2* was the most upregulated ISG, particularly in OSF transduced with rSeV-GFP-059, followed by *A3Z1* and *SAMHD1*. These ISGs are known to play critical roles in antiviral defense against SRLV [50,54,55], and their upregulation suggests that SeV vectors could enhance the antiviral state of host cells beyond ORFV-specific immunity. Notably, rSeV-GFP-059 induced a statistically significant increase in *OBST2* expression at 48 hpt compared to the other vectors, suggesting an important role for *ORFV 059* in stimulating *OBST2*. Although no direct evidence currently links *OBST2* to ORFV infection, its well-established antiviral activity against lentiviruses and other enveloped viruses suggests that *OBST2* may play a role in restricting ORFV replication [73]. However, vaccinia virus has been shown to be resistant to BST2-mediated restriction, likely due to its cytoplasmic replication and unique egress mechanisms [74]. Nonetheless, the upregulation of *OBST2* observed in our study may reflect a broader activation of the type I IFN responses, and its potential role in modulating immunity against ORFV remains to be explored. In addition to these observations, the stronger innate activation induced by rSeV-GFP-059 compared to both the empty SeV vector and rSeV-GFP-B2L suggests that the enhanced response cannot be attributed solely to the SeV backbone, as stated before, against SRLV [50]. Although SeV inherently stimulates antiviral signaling, the expression of ORFV 059 appears to potentiate this effect. ORFV 059 is an immunogenic envelope protein involved in early virus–host interactions, and its expression within an RNA viral vector may generate additional PAMPs or modulate intracellular sensing pathways, thereby amplifying RIG-I and OBST2 activation [42]. While the precise molecular mechanism remains to be elucidated, these findings indicate that ORFV 059 contributes directly to innate immune stimulation, rather than acting as a passive transgene. Future studies will focus on dissecting these mechanisms and determining how ORFV 059 enhances antiviral signaling in vivo.

In addition to the upregulation of innate immune-related genes, antiviral activity assays demonstrated a pronounced antiviral effect of rSeV-GFP-059. A substantial reduction in ORFV DNA copies was observed as early as 24 hpi in rSeV-GFP-059-transduced cells, reaching statistical significance at 5 dpi, compared with the positive control. Furthermore, although no significant differences were observed, ORFV-specific RNA transcription was diminished at both 24 and 48 hpi in rSeV-GFP-059-transduced KOP-R cells, indicating a modest early suppression of viral gene expression. Notably, CPE was absent in KOP-R cells transduced with rSeV-GFP-059 compared to all other groups at 5 dpi. These results suggest that rSeV-GFP-059 not only activates the antiviral signaling pathways, but also establishes a sustained protective state that limits ORFV replication and associated cellular damage. Importantly, supernatants from OSF transduced with SeV vectors exhibited antiviral activity against SRLV, as evidenced by reduced viral DNA copy numbers. In contrast to the ORFV model, this antiviral effect was most prominent with both SeV-GFP and rSeV-GFP-B2L, significantly decreasing the SRLV load. However, SeV-GFP did not induce *IFN-β* expression, suggesting that the observed antiviral activity is not solely mediated by IFN-β. Other cytokines, such as other type I interferons (e.g., IFN-α) [75], type II interferons (e.g., IFN-γ), IFN-λ, and additional pro-inflammatory cytokines [64], may also contribute to the observed antiviral response. Further studies are warranted to elucidate which specific interferons or cytokines are involved in the innate immune responses triggered by SeV vectors.

Although IFN-β and ISG induction was assessed at the transcriptional level in this study, the strong antiviral phenotype observed suggests that multiple innate immune pathways are being activated beyond the specific cytokines quantified. Transcriptional upregulation of IFN-β and ISGs is a well-established early marker of antiviral signaling, and the consistent reduction in ORFV and SRLV replication indicates that these pathways are functionally engaged [54,70]. Nevertheless, we acknowledge that protein-level confirmation of cytokine secretion would further strengthen the mechanistic interpretation. Future studies will incorporate cytokine profiling and proteomic analyses to determine the relative contribution of IFN-β, OBST2, A3Z1, SAMHD1 and other innate mediators to the antiviral state induced by the recombinant SeV vectors.

In summary, these findings support the use of recombinant SeV vectors as promising platforms for the development of novel vaccines against ORFV. The ability of rSeV-GFP-059 to achieve efficient transgene expression, stimulate innate immune responses, and exert antiviral effects against ORFV in ovine cells underscores its potential for further in vivo evaluation.

Finally, it is important to acknowledge the limitations of this study. First, the assessment of innate immune activation was primarily based on transcriptional profiling. While the robust upregulation of mRNA provides strong evidence of pathway induction, correlating these results with protein-level quantification via ELISA or Western blot would offer a more comprehensive functional validation of cytokine secretion. Second, although our in vitro models demonstrated significant antiviral activity and immune stimulation, these systems cannot fully recapitulate the systemic complexity of the host immune response. Consequently, in vivo challenge studies in the natural host are essential and are currently required to definitively establish the safety, immunogenicity, and protective efficacy of the rSeV-GFP-059 vector against ORFV infection.

## 5. Conclusions

In conclusion, this study demonstrates the feasibility of using recombinant SeV vectors as a versatile vaccine platform against ORFV. Notably, rSeV-GFP-059 achieved significant transgene expression, robust *RIG-I* upregulation, and induction of *IFN-β* and *OBST2*. These responses correlated with a marked reduction in ORFV DNA copies, a slight decrease in RNA replication and the prevention of cytopathic effects. These findings suggest that *ORFV 059* may confer intrinsic immunostimulatory advantages, enhancing the antiviral state beyond ORFV-specific immunity. Furthermore, the secretion of soluble antiviral factors that are capable of inhibiting heterologous viruses such as SRLV highlights the broader immunomodulatory potential of SeV vectors. Collectively, our results establish rSeV-GFP-059 as a promising candidate for further in vivo evaluation in sheep and goats, supporting the development of novel SeV-based vaccines for veterinary applications and expanding their potential as platforms for delivering heterologous antigens.

## Data Availability

All data are available in this manuscript.

## Supplementary Materials

The following supporting information can be downloaded at: https://www.mdpi.com/article/10.3390/v18040462/s1, Supplementary Table S1: Primers and probe sequences used in the study.

## Abbreviations

The following abbreviations are used in this manuscript:

A3Z1	Apolipoprotein B mRNA editing enzyme catalytic subunit 3Z1
ANOVA	One-way analysis of variance
ASR	Adjusted standardized residuals
CE	Contagious ecthyma
CPE	Cytopathic effect
DMEM	Dulbecco’s modified Eagle’s medium
DNA	Deoxyribonucleic acid
dpi	Days post-infection
dsDNA	Double-stranded deoxyribonucleic acid
dsRNA	Double-stranded ribonucleic acid
ELISA	Enzyme-linked immunosorbent assay
FBS	Fetal bovine serum
GFP	Green fluorescent protein
hpi	Hours post-infection
hpt	Hours post-transduction
ITRs	Inverted terminal repeats
IFN	Interferon
IFN-β	Interferon beta
ISGs	Interferon-stimulated genes
KOP-R	Bovine esophagus cells
MOI	Multiplicity of infection
mRNA	Messenger ribonucleic acid
MyD88	Myeloid differentiation primary response 88
OBST2 (BST2)	Ovine bone marrow stromal antigen 2 (Tetherin)
ORFV	Orf virus
OSF	Ovine skin fibroblasts
PCR	Polymerase chain reaction
PRRs	Pattern recognition receptors
qPCR	Quantitative polymerase chain reaction
RIG-I	Retinoic acid-inducible gene I
SAMHD1	SAM and HD domain-containing protein 1
SD	Standard deviation
SeV	Sendai virus
SRLV	Small ruminant lentiviruses
ssRNA	Single-stranded ribonucleic acid
TCID_50_/mL	50% tissue culture infectious dose per milliliter
TLR	Toll-like receptor
TLR2, TLR3, TLR4, etc.	Toll-like receptor 2, 3, 4… (as indicated)
5′ppp	5′-triphosphate
