# Supplementary material for: In Vitro Antiviral Properties of Two Recombinant Sendai Virus Vectors Encoding ORFV 011 and ORFV 059 Genes
Source: Viruses. 2026 Apr 13;18(4):462. doi: 10.3390/v18040462 (PMC13119788; doi:10.3390/v18040462)
Supplement: Supplementary file 1 [file viruses-18-00462-s001.zip › viruses-4248374-supplementary.pdf]

Supplementary Materials:

Supplementary Table S1. Primers and probe sequences used in the study.

Name	Primer sequence (5´–3´)	Descriptions
**ORFV 045**	Fw: CCTACTTCTCGGAGTTCAGC	Used in ORFV infection diagnosis in sheep and antiviral activity assay
	Rv: GCAGCACTTCTCCTCGTAG	
**ORFV B2L A**	Fw: GTCGTCCACGATGAGCAGCT	Used in amplification from NAV
	Rv: TACGTGGGAAGCGCCTCGCT	
**ORFV 059 A**	Fw: ACAGCTGGATCCACCCGAAATCACGGC	Used in amplification from NAV
	Rv: AGAATTCTCACACGATGGCCGTGACCAG	
**B2L NotI IF**	Fw: GTGGTGACAGCGGCCGCATGTGGCCGTTCTCCTCCA	Used to clone ORFV B2L into SeV-GFP
	Rv: TCCGGATCCGCGGCCGCTTTTATTTATTGGCTTGCAGAACTCCGAGC	
**059 NotI IF**	Fw: GTGGTGACAGCGGCCGCATGGATCCACCCGAAATCACG	Used to clone ORFV 059 into SeV-GFP
	Rv: TCCGGATCCGCGGCCGCTTTTCACGATGGCCGTGACC	
**ORFV B2L B**	Fw: CGTGATCATTACCGAGTGGAAG	Used in transgene expression
	Rv: GTCCACGATGAGCAGCTTAGTG	
**ORFV 059 B**	Fw: AGCTATACGACGCGATCATCAA	
	Rv: CCGCGTCTTCACCTGTATGTAG	
**TLR1**	Fw: CCCACAGGAAAGAAATTCCA	Used in gene expression
	Rv: GGAGGATCGTGATGAAGGAA	
**TLR2**	Fw: CCGAAAGCACAAAGATGGTT	
	Rv: ACGACGCCTTTGTGTCCTAC	
**TLR3**	Fw: GAGGCAGGTGTCCTTGAACT	
	Rv: GCTGAATTTCTGGACCCAAG	
**TLR4**	Fw: TGGATTTATCCAGATGCGAAA	
	Rv: GGCCACCAGCTTCTGTAAAC	
**TLR5**	Fw: CATCAGATGGAACTGGGACA	
	Rv: AAAACCACATCGCCAACATC	
**TLR6**	Fw: GTTTTCCCAGTCACGAC	
	Rv: CAAAGCAGGGAACAATCCAT	
**TLR7**	Fw: GCTGGAGAGATGCCTGCTAT	
	Rv: ACTCCTTGGGGCTAGATGGT	
**TLR8**	Fw: GGTCCCAATCCCTTTCCTCTA	
	Rv: TCCACATCCCAGACTTTCTACGA	
**TLR9**	Fw: CACCTCCGTGAGGTTGTTGT	
	Rv: CTCGTATCCCTGTCGCTGAG	
**TLR10**	Fw: TCTGCCTGGGTGAAGTATGA	
	Rv: AATGGCACCATTCAGTCTGG	
**RIG-I**	Fw: GCTGACGGCCTCAGTTGGT	

	Rv: TCGAGAGAAGCACACAGTCTGC	
**MyD88**	Fw: GCATCGAGGAGGACTGCCA	
	Rv: CCGAGGGATGCTGCTGTCT	
**IFN-B**	Fw: GATGCCGTATTGGTCATGTA	
	Rv: CATCTGCCCATAGAGTTCCT	
**A3Z1**	Fw: TCCGTTCTTGGAATCTGGAC	
	Rv: GTATAGATGCGGGAGGCAAA	
**OBST2**	Fw: CGTGGACGGCCTCCAAG	
	Rv: TGGCAGCTTCGGCTTCC	
**SAMHD1**	Fw: GAGAACGAAGCTGCTTAATTGTATCC	
	Rv: GAGGTGTGTCGATGATTCGGA	
**B-actin**	Fw: CTCACGGAGCGTGGCTACA	Used in transgene and gene expression
	Rv: TACGTGGGAAGCGCCTCGCT	
**MVV**	Fw: CTCCTTGCAGGCCACAATG	Used in antiviral activity assay
	Rv: GCTGCTTGCACTGTCTCGG	
**MVV probe**	6-FAM-TGCCTTATGTGTAGTCAGC-TAMRA	
